# Targeted Deletion of the *Claudin12* Gene in Mice Increases Articular Cartilage and Inhibits Chondrocyte Differentiation

**DOI:** 10.3389/fendo.2022.931318

**Published:** 2022-07-22

**Authors:** Weirong Xing, Sheila Pourteymoor, Yian Chen, Subburaman Mohan

**Affiliations:** ^1^ Musculoskeletal Disease Center, VA Loma Linda Healthcare Systems, Loma Linda, CA, United States; ^2^ Department of Medicine, Loma Linda University, Loma Linda, CA, United States; ^3^ Department of Biochemistry, Loma Linda University, Loma Linda, CA, United States; ^4^ Department of Orthopedic Surgery, Loma Linda University, Loma Linda, CA, United States

**Keywords:** *Claudin12*, knockout, bone, chondrocyte, cartilage, chondrocyte differentiation

## Abstract

To study the role of Claudin (CLDN)12 in bone, we developed mice with a targeted deletion of exon2 in the *Cldn12* gene for skeletal phenotype analysis. Micro-CT analysis of the secondary spongiosa of distal femurs of mice with targeted disruption of the *Cldn12* gene and control littermates showed no significant genotype-specific differences in either cortical or trabecular bone parameters for either gender in 13-week-old mice. Immunohistochemistry revealed that while CLDN12 was expressed in both differentiating chondrocytes and osteoblasts of the secondary spongiosa of 3-week-old wild-type mice, its expression was restricted to differentiating chondrocytes in the articular cartilage and growth plate in adult mice. Articular cartilage area at the knee were increased by 47% in *Cldn12* knockout (KO) mice compared to control littermates. Micro-CT analyses found that while the trabecular number was increased by 9% and the trabecular spacing was reduced by 9% in the femoral epiphysis of *Cldn12* KO mice, neither bone volume nor bone volume adjusted for tissue volume was different between the two genotypes. The expression levels of *Clusterin*, *Lubricin* and *Mmp13* were increased by 56%, 46%, and 129%, respectively, in primary articular chondrocytes derived from KO compared to control mice. Our data indicate that targeted deletion of the *Cldn12* gene in mice increases articular cartilage, in part, by promoting articular chondrocyte phenotype.

## Introduction

The claudin (CLDN) family comprises 24 members of 20–34 kDa tetraspan transmembrane proteins in tight junctions (1, 2). Sequence analyses and structural comparison of claudins has led to classification of two groups, the canonical claudins (1–10, 14, 15, 17, 19) and the non- canonical claudins (11–13, 16, 18, 20–24) (1). Since claudins polymerize to form tight junctions, their canonical functions include regulation of paracellular transport of ions, small molecules, and water, as well as maintenance of the distribution of lipids and proteins in the apical and basal regions of the plasma membrane (1, 3). However, there is increasing evidence that some claudins have additional non-canonical functions in which they participate in intracellular signaling by phosphorylation of its specific amino acids in PDZ binding motifs of the cytoplasmic domain by Rho kinase, which allow them to interact with other proteins (4–6). While several claudins are expressed in bone, nothing is known about their function in regulation of bone homeostasis before recent studies in our laboratory demonstrated that CLDN18 was a novel regulator of bone resorption. Disruption of the *Cldn18* gene in mice reduced trabecular bone mass at multiple skeletal sites due to increased osteoclast formation and bone resorption (7). Evaluation of expression levels of claudins during osteoblast differentiation revealed that osteoblasts express multiple *Claudins* of which *Cldn11* expression was the most regulated during vitamin C-induced differentiation of osteoblasts, *in vitro* (8). Mice with disruption of *Cldn11* exhibited a low bone mass phenotype. Trabecular bone mass of the femur of the *Cldn11* knockout (KO) mice was reduced by 40% that was primarily caused by reduced osteoblast differentiation and impaired bone formation (9). Loss of *Cldn3* in mice disrupted tight junctions and impaired amelogenesis (10). Mice lacking *cldn16* displayed magnesium and calcium wasting whereas absence of *Cldn10* (*from Henle’s loop*) resulted in hypermagnesemia and interstitial nephrocalcinosis (11, 12). These studies suggest that CLDN functions are tissue- and cell-type specific.

Our previous studies demonstrated that expression levels of both classic and non-classic claudins are regulated during RANKL-induced osteoclast differentiation. In these studies, we found that while *Cldn12* expression was upregulated during osteoclast differentiation, there was a dramatic inhibition of *Cldn12* expression during osteoblast differentiation (8). Since nothing was known regarding the role of CLDN12 in the musculoskeletal tissues, we evaluated the role of CLDN12 by generating mice with targeted disruption of the *Cldn12* gene in all cells and evaluated its skeletal and cartilage phenotypes by established methods. Our findings demonstrated a novel role for *Cldn12* in regulating articular chondrocyte phenotype.

## Materials and Methods

### *Cldn12* KO Mice

The *Cldn12^tm1(KOMP)Vlcg^* mouse strain used for this research project was created from ES cell clone 13208A-A6, generated by Regeneron Pharmaceuticals, Inc., and made into live mice by the KOMP Repository (www.komp.org) and the Mouse Biology Program (www.mousebiology.org) at the University of California Davis (Davis, CA, USA). Disruption of *Cldn12* gene resulted in replacement of the *Cldn12* open reading frame with *LacZ* expression cassette. Brain tissue derived from *Clnd12* KO mice expresses LacZ as reported (13). Homozygous *Cldn12* KO male mice were bred with heterozygous females to obtain homozygous KO and littermate heterozygous controls for skeletal phenotype evaluation. Mice were genotyped using PCR and euthanized at 13 weeks of age for skeletal phenotype evaluation. In our preliminary studies, we found that *Cldn12* expression in bones were not different between the wild-type and heterozygous mice. We, therefore, chose to breed homozygous heterozygous male mice with heterozygous female mice in order to generate more mice efficiently for our experiments (50% heterozygous and 50% knockout mice) and used heterozygous mice as controls. Animals were housed at the Veterans Administration Loma Linda Healthcare System (VALLHS) according to approved standards with controlled temperature (22°C) and illumination (14-h light, 10-h dark), as well as unlimited food and water. Animal procedures were performed by a protocol (MHO0007/00014) approved by the Institutional Animal Care and Use Committee of the VALLHS. Mice were anesthetized with anesthetics (isoflurane) prior to the procedures. The animals were euthanized by exposure to carbon dioxide followed by cervical dislocation.

### Micro-CT Evaluation

Femurs and tibias isolated from 13-week-old- mice were scanned by X-ray at 55 kVp volts to measure trabecular bone parameters at a resolution of 10.5 µm/slice. Trabecular bone parameters were measured at the epiphysis and the secondary spongiosa region of the distal femur using micro-computed tomography (microCT, Scanco vivaCT40, SCANCO Medical AG, Zurich, CH-8306, Switzerland). The trabecular bone of the secondary spongiosa region started at 0.36 mm from the distal growth plate in the direction of the metaphysis and extended for 2.25 mm. Cortical bones at mid-diaphysis of the femur were scanned at 70 kV energy, and 114 µA intensity. Data were quantified from 200 slices (2.1 mm) of cortical bone. The exact numbers and location of slices used for analyses were adjusted for length so that the analyzed regions were anatomically comparable between samples. Bone volume (BV, mm^3^), bone volume fraction (BV/TV, %), trabecular number (Tb. N, mm^-1^), trabecular thickness (Tb. Th, mm) and trabecular space (Tb. Sp, mm) were evaluated as reported (14–16).

### Histology and Immunohistochemistry

The articular cartilage phenotype of heterozygous and mutant male and female mice were evaluated at the femoral and tibial aspects of the knee joint. The knee joints were fixed in 10% formalin, decalcified in 20% EDTA at 4 ^0^C for 4 weeks, dehydrated, and embedded in paraffin. The knee was first cut to middle of the bone and then sectioned at a thickness of 4 µm and stained every 5^th^ slide with Safranin-O using standard protocols. Articular cartilage area and width were quantitated (5 sections per knee joint) by a blinded observer using the OsteoMeasure V3.1.0.2 computer software. (OsteoMetrics, Decatur, GA) (17, 18). Epiphysis sections of the distal femur were stained with trichrome for histomorphometry analyses as reported (19). Trabecular bone parameters of the secondary ossification center of the epiphysis and the secondary spongiosa were measured in a blinded fashion with computer software OsteoMeasure as described (17, 18).

### Immunohistochemistry

Immunohistochemistry was performed using a rabbit immunohistochemistry kit (Vector Laboratories, Burlingame, CA). Briefly, femoral sections were de-paraffinized in histochoice clearing agent, rehydrated in a graded series of ethanol and tap water, and treated with 3% H_2_O_2_ for 30 minutes to inactivate endogenous peroxidase activity. The sections were then rinsed with PBS (pH 7.4) and heated for 20 minutes at 90 ^0^C in sodium citrate citric acid buffer (pH2.5) for epitope recovery. The sections were pretreated with a blocking solution containing normal goat serum for 20 minutes, and then incubated with anti-Claudin 12 rabbit polyclonal antibody (Cat NO: SKU 18801, Immuno-Biological Laboratories, Inc. Minneapolis, MN 55432) at a concentration of 25 μg/ml. Negative control sections were incubated with normal rabbit IgG. After an overnight incubation at 4 ^0^C, the sections were rinsed with PBS, and incubated with biotinylated secondary antibodies for 30 minutes at room temperature. The slides were then washed in PBS, incubated with the VECTASTAIN ABC-AP kit (Cat No AK-5000, Vector Laboratories) for 30 minutes, rinsed again with PBS, and incubated with the Vector Blue substrate until the desired blue color stain developed.

### Primary Articular Chondrocyte Culture, RNA Extraction and RT-Real Time PCR

Primary articular chondrocytes were isolated from the articular cartilage of the distal femoral epiphyses and proximal tibial epiphyses of 3-day old C57BL/6 mice (4 pairs of female and male mice) and cultured as previously described (20, 21). Cells were grown in DMEM/F12 medium containing 10% fetal bovine serum (FBS), penicillin (100 U/mL), and streptomycin (100 μg/mL) to approximately 75% confluence for RNA extraction. Total RNA was extracted from chondrocytes with the Trizol reagent as described previously (22, 23). An aliquot of RNA (300 ng) was reverse-transcribed into cDNA in a 20 µl volume using an oligo(dT)_12-18_ primer. The real time PCR reaction mix contained 0.5 µl of template cDNA, 1x SYBR GREEN master mix (Cat No 204143, Qiagen), and 100 nM of the specific forward and reverse primers in a 25 μl reaction volume. Primers used for real-time PCR are listed in **Table 1**. Relative gene expression was determined by the ^ΔΔ^CT method (24).

**Table 1:** Primer sequences used for real time PCR and genotyping.

Gene	Forward primer	Reverse primer
*Ppia*	5’-CCATGGCAAATGCTGGACCA	5’-TCCTGGACCCAAAACGCTCC
*Cldn12*	5’- ACTGCTCTCCTGCTGTTCGT	5’-TGTCGATTTCAATGGCAGAG
*Cldn11*	5;-CTGCCGAAAAATGGACGAACTG	5’-TGCACGTAGCCTGGAAGGATG
*Cldn 18*	5’-GCTGTACGAGCCCTGATGAT	5’-GAGATGATGAACAAGATCCC
*Col2*	5’- TGGCTTCCACTTCAGCTATG	5’- AGGTAGGCGATGCTGTTCTT
*Col10*	5’- ACGGCACGCCTACGATGT	5’- CCATGATTGCACTCCCTGAA
*Acan*	5’- GACCAGGAAGGGAGGAGTAG	5’- CAGCCGAGAAATGACACC
*Sox9*	5’- CGGAGGAAGTCGGTGAAGA	5’- GTCGGTTTTGGGAGTGGTG
*MMP13*	5’-CATCCATCCCGTGACCTTAT	5’-TCATAACCATTCAGAGCCCA
*Clusterin*	5’-AGAAGGTGAAGATGACCGCA	5’-CTTGTACTGCTCTGTCAGCC
*Lubricin*	5’-TGGATGGACTGACTACGCTG	5’-CGGTAATTCTGCGTGGTGGA
*Cldn12 WT allele*	5’-CGGCTCAAACTTCCTGTTGAGAT	5’AACATCAAACTGGCCAAGTGTCT
*Clden12 Mt allele*	5’-GCAGCCTCTGTTCCACATACACT	5’ACAGACAAACTCCTAGCCTCATC

### Statistical Analysis

Data are presented as mean ± SEM from 8 (4 male and 4 female mice) replicates per group. Data were analyzed by a Student’s t-test or two-way ANOVA analysis as appropriate.

## Results

### Mice With Disruption of the *Cldn12* Gene Exhibit No Changes in Body Weight, Body Length and Femur Length

**Figure 1:**
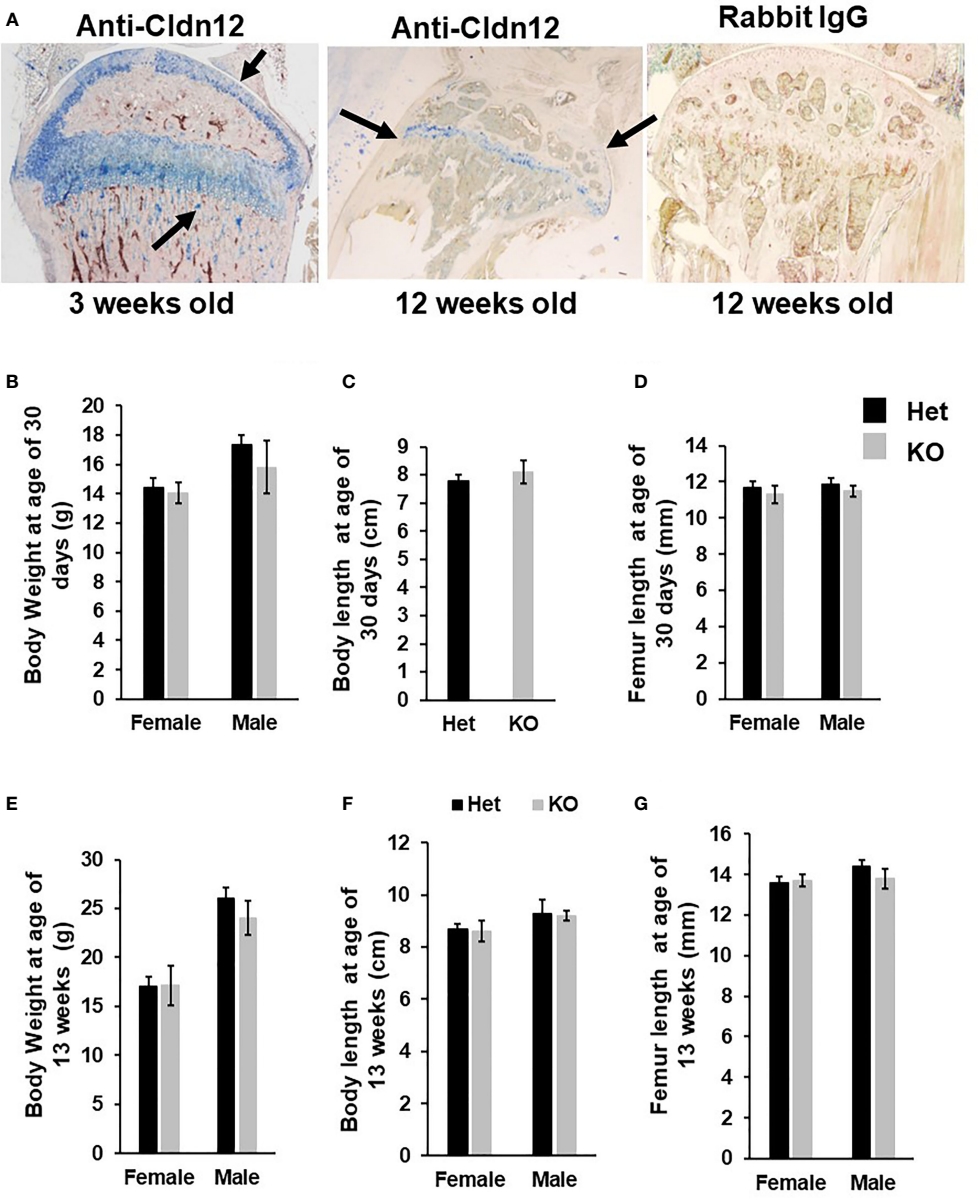
Mice with disruption of *Cldn12* exhibit no changes in body weight, body length and femur length at ages of 30 days and 13 weeks. **(A)** Expression of CLDN12 in chondrocytes and osteoblasts in the tibia of 3- and 12-weeks old mice, detected by immunohistochemistry. Arrows indicate blue staining in CLDN12 positive cells. **(B-D)** Body weight, body length, and femur length of the *Cldn12* knockout (KO) and the control heterozygous littermates at 30 days of age, respectively. **(E-G)** Body weight, body length, and femur length of the *Cldn12* knockout (KO) and the control littermates at 13 weeks of age, respectively.

**Figure 2:**
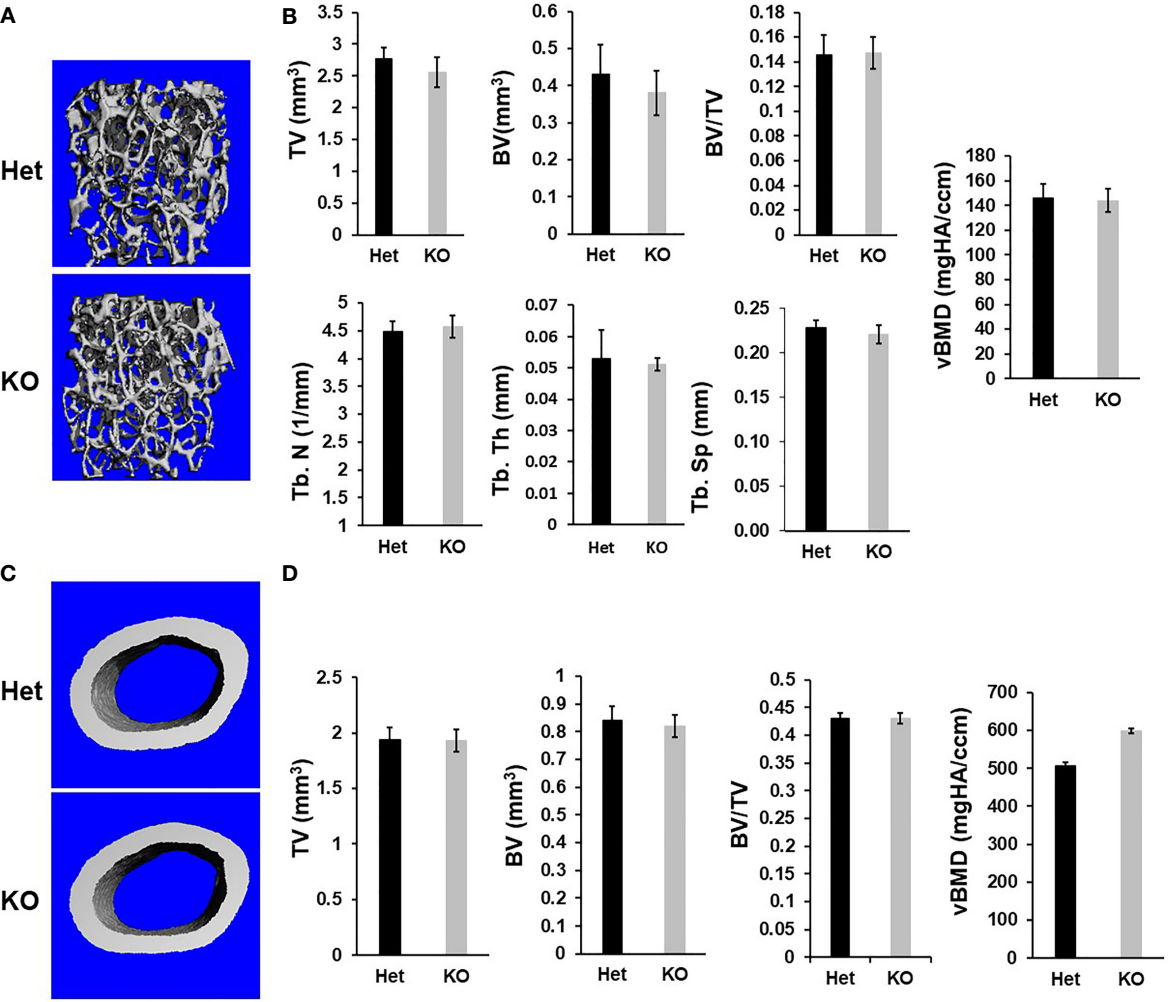
Micro-CT analyses reveals no changes in trabecular and cortical parameters of the femur of the *Cldn12* cKO mice at 13 weeks of age. **(A)** Micro-CT images of the trabecular bone of the distal femur of the KO and control heterozygous mice. **(B)** Quantitative micro-CT data of the trabecular bone of the distal femur. **(C)** Micro-CT images of the cortical bone of the KO and control heterozygous mouse femurs. **(D)** Quantitative micro-CT data of the cortical bone of the femur. TV, tissue volume; BV, bone volume; Tb. N, trabecular number; Tb.Th, trabecular thickness; Tb.Sp, trabecular spacing; vBMD, volumetric bone mineral density. Values are Mean ± SEM (n=8, 4 males and 4 females).

Immunohistochemistry revealed that while *Cldn12* is expressed in both differentiating chondrocytes and osteoblasts of the secondary spongiosa of 3-week-old wild-type mice, its expression is restricted to differentiating chondrocytes of the articular cartilage and growth plate in 12-week-old C57BL/6 adult mice (**Figure 1A**). Since *Cldn12* was highly expressed in the long bones, we examined the body weight, body length and femur length of the *Cldn12* KO mice. We found that body weight, body and femur length were not significantly different in either male or female *Cldn12* KO mice at 30 days and 13 weeks of age compared to corresponding gender matched control heterozygous littermates (**Figures 1B–G**). Since in our preliminary analyses we did not find a significant difference in trabecular bone volume between the two genotypes in either gender and since we used similar number of male and female mice, we opted to use a mixed gender data in order to increase the power for this communication for micro-CT and histology analyses. MicroCT analysis of mouse femurs with targeted disruption of the *Cldn12* gene and control littermates showed no significant genotype-specific differences in trabecular parameters of TV, BV, BV/TV, trabecular number, thickness and spacing and vBMD of the gender-mixed 13-week-old mice (**Figures 2A, B**). There were also no changes in the cortical bone parameters of TV, BV, BV/TV and vBMD in these mice (**Figures 2C, D**).

### Targeted Deletion of *Cldn12* in Mice Increases Articular Cartilage and Trabecular Bone of the Epiphysis but Inhibits Chondrocyte Differentiation

**Figure 3:**
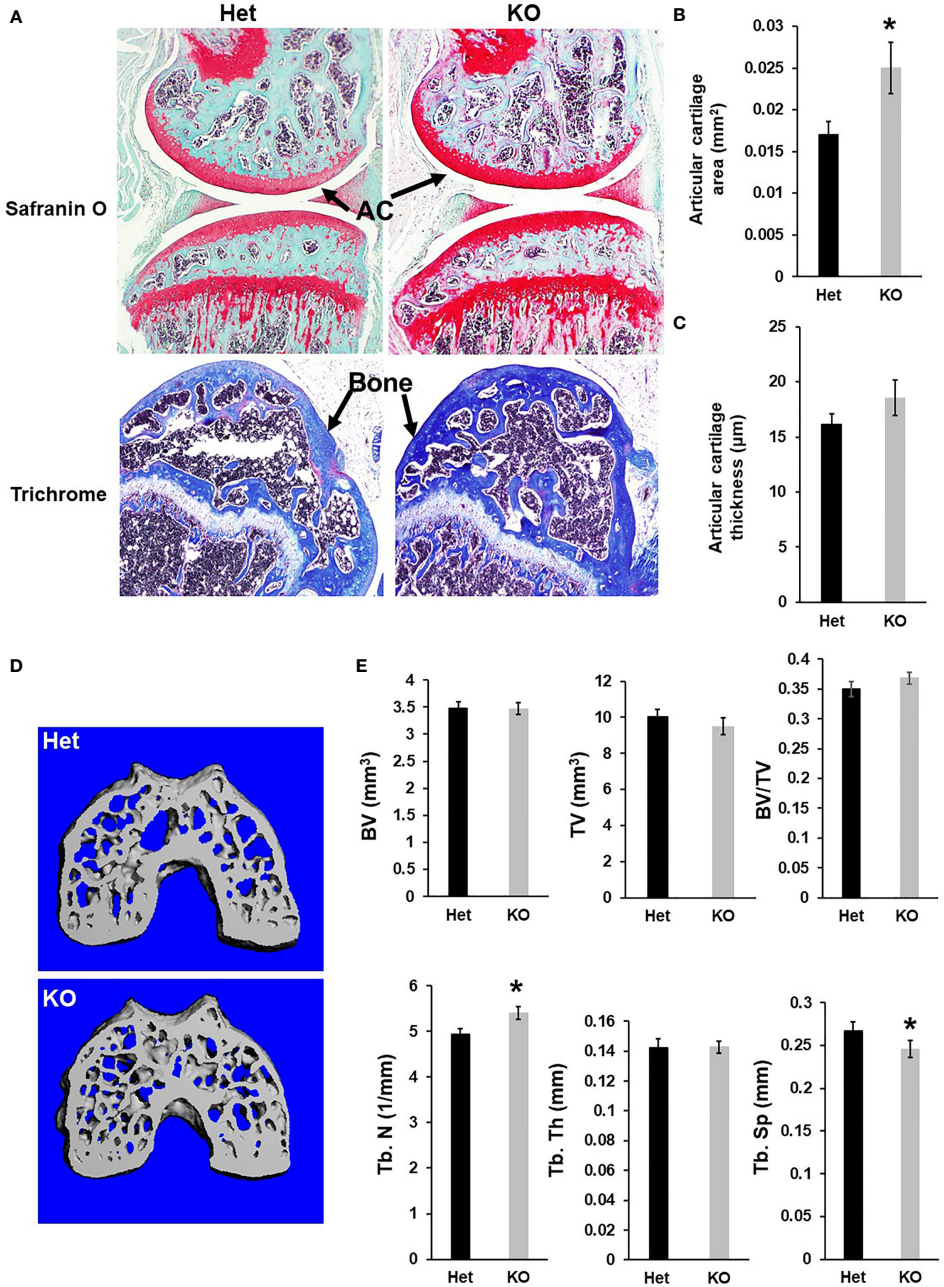
Mice with disruption of *Cldn12* exhibit increased articular cartilage area of the distal femur. **(A)** Longitudinal section images of the knees of the *Cldn12* KO and control heterozygous mice, stained with safranin orange. The distal femoral epiphyses of the *Cldn12* KO and control heterozygous mice were stained with trichrome blue and counter-stained with safranin orange. **(B, C)** Quantitative histomorphometry data of the articular cartilage area and thickness of the knee, respectively. **(D)** Micro-CT images of the cress sections of the distal femoral epiphyses. **(E)** Quantitative micro-CT data of the trabecular bone parameters of the distal femoral epiphyses, respectively. TV, tissue volume; BV, bone volume; Tb. N, trabecular number; Tb.Th, trabecular thickness; Tb.Sp, trabecular spacing. Values are Mean ± SEM (n=8). A star (*) indicates P<0.05, as compared to the control littermates.

**Figure 4:**
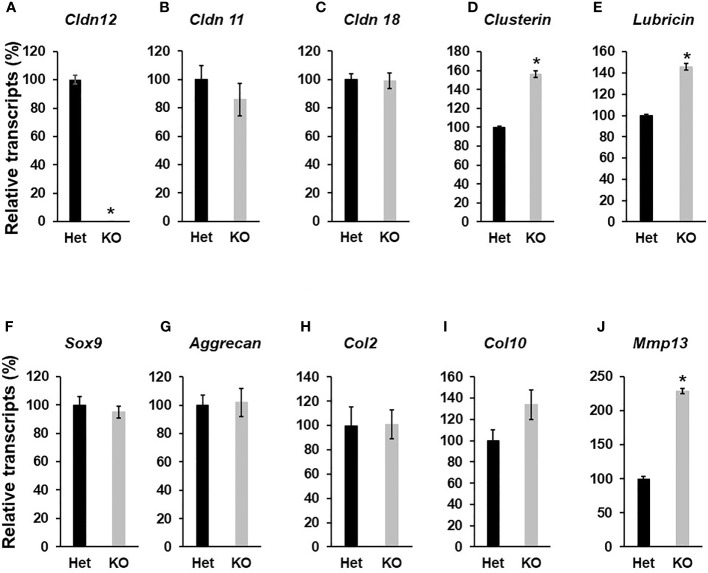
Disruption of *Cldn12* inhibits chondrocyte differentiation. Primary articular chondrocytes were isolated from 3 days of the epiphyses of the femur and tibia of newborns. Cells were cultured up to 70% confluency, followed by RNA extraction. Total RNA was reversely transcribed into cDNA for quantitative real-time PCR. **(A–E)** Expression levels of *Cldn12, Cldn 11*, *Cldn 18*, *Clusterin*, and *Lubricin*, respectively. **(F–J)** Expression levels of *Sox9, Aggrecan*, and *Collagen 2α1 (Col2), Collagen 10α1 (Col10)*, *and Mmp13*, respectively. Values are Mean ± SEM (n=4). Star indicates P<0.05, as compared to the control cells derived from heterozygous control mice. A star (*) indicates P<0.05, as compared to the control littermates.

Since *Cldn12* was highly expressed in articular chondrocytes, besides osteoblasts, we next evaluated the articular cartilage and epiphysis phenotype of 13-week-old *Cldn12* KO and control mice. Articular cartilage area at the knee was increased by 47% in gender mixed *Cldn12* KO mice compared to gender matched controls (**Figures 3A, B**). There was an observed tendency to increased articular cartilage thickness but no increase in the *cldn12* KO mice (**Figure 3C**). Micro-CT analyses indicated that the trabecular number of the femoral epiphysis was increased by 9% while the trabecular spacing was reduced by 9% in the *cldn12* KO mice compared to the littermate controls (**Figures 3D, E**). There were no significant differences in the TV, BV, BV/TV and trabecular thickness between the *cldn12* KO and control mice. To determine if *Cldn12* regulates chondrocyte differentiation, we measured marker gene expression for osteoblast and chondrocyte differentiation in primary articular chondrocytes. As expected, expression of *Cldn12* in the articular chondrocytes derived from the *Cldn12* KO mice was not detectable (**Figure 4A**). The expression level of neither *Cldn11 nor Cldn 18* was significantly altered in the articular chondrocytes of *Cldn12* KO mice compared to control mice (**Figures 4B, C**). The expression levels of *Clusterin* and *Lubricin*, markers of articular chondrocytes, were increased by 56% and 46%, respectively in primary cultures of articular chondrocytes derived from KO mice compared to control mice (**Figures 4D, E**). Of the established markers of chondrocyte differentiation, expression levels of *Sox9*, *Aggrecan, Col2α1* and *Col10α1* were not altered between the two genotypes (**Figures 4F–I**). However, the expression level of *Mmp13* was elevated by 129% in the articular chondrocytes derived from *Cldn12* KO mice compared to control mice (**Figure 4J**).

## Discussion

CLDN12, a member of the claudin family, was thought to function as an integral membrane protein and component of tight junction complexes to serve as a physical barrier to prevent solutes and water from passing freely through the paracellular space between epithelial or endothelial cell sheets (25). The tight junction complexes may also play critical roles in maintaining cell polarity and signal transductions. Unlike CLDN18, however, CLDN12 is an atypical member of the claudin family because it does not have a PDZ binding motif that can bind to the intracellular scaffolding proteins ZO-1, ZO-2 and ZO-3 and mediate an interaction with the cytoskeleton (26). Although an *ex vivo* study found that calcium permeability was reduced in the renal proximal tubules derived from *Cldn12* KO mice, the urinary calcium excretion was not altered in the KO mice including those on different calcium containing diets (27). The role of CLDN12 in regulating bone mineralization in bone and cartilage tissues has not been characterized yet. In this study, we report that CLDN12 was expressed in both differentiating chondrocytes and osteoblasts of the secondary spongiosa of 3-week-old wild-type mice. Its expression was found to be more restricted to differentiating chondrocytes of the articular cartilage and growth plate than to osteoblasts in the trabecular bone of adult mice. It is known that SOX9 maintains growth plate and articular cartilage healthy by preventing chondrocyte dedifferentiation followed by redifferentiation into osteoblasts (28). Estrogen has been implicated in the acceleration of the programmed senescence of the growth plate which may in part be mediated *via* inhibiting Sox9 expression (29, 30). Thus, the issue of whether the difference in CLDN12 expression is caused by changes in expression levels of Sox9 seen in prepubertal and adult mice remains to be established. In this regard, there are published data to indicate that Sox9 regulates claudin expression in other cell types (31).

Consistent with the lack of detectable *Cldn12* expression in bone cells of adult mice, we found that none of the trabecular or cortical bone parameters measured showed significant change between the two genotypes at the secondary spongiosa of distal femur. While the trabecular number was increased by 9% and trabecular separation was decreased by 9% at the epiphysis of *Cldn12* KO mice, there was no corresponding change in either trabecular bone volume or trabecular bone volume adjusted for tissue volume at this site between the two genotypes. The body weight, body length and femur length of the KO mice were comparable to the gender-matched littermate control in 30-day and 13-week-old mice in both genders. However, we observed an increase in articular cartilage area and a slight increase in articular cartilage width at the knee in *Cldn12* KO mice compared to littermate controls. *In vitro* studies found that the lack of CLDN12 in articular chondrocytes caused an increased expression of articular chondrocyte markers, *Clusterin and Lubricin*, thus suggesting CLDN12 inhibits articular chondrocyte proliferation/maintenance. Surprisingly, expression levels of *Mmp13*, known to be involved in articular cartilage degradation, was increased in the Cldn12 KO mice compared to control heterozygous mice. Our data indicate that targeted deletion of the *Cldn12* gene in mice increases articular cartilage, in part, by promoting the articular chondrocyte phenotype.

By contrast to the lack of skeletal phenotype in *Cldn12* KO mice, trabecular bone mass was significantly decreased in mice with disruption of *Cldn11* or *Cldn18* genes in mice. The lack of a skeletal bone phenotype in *Cldn12* KO mice compared to *Cldn11* and *Cldn18* KO mice could be explained by a possible compensation by other members of CLDN family proteins. We showed that expression levels of neither claudin-11 nor claudin-18 were significantly altered in the articular chondrocytes of *Cldn 12* KO mice compared to control mice. It has been reported that *Cldn2* and *Cldn12* double KO mice displayed reduced intestinal calcium absorption and reduced colonic calcium permeability (32). The double KO mice exhibited significantly greater urinary calcium wasting than *Cldn2* null mice, hypocalcemia and reduced BMD which was not observed in single-KO mice (32). It is also known that the reduced expression of *Cldn14* in the cortical thick ascending limb, where it serves as a negative regulator of calcium permeability could rescue the functional loss of *Cldn12* in the renal proximal tubule in *Cldn12* KO mice (27). The mechanisms by which *Cldn12* regulates downstream signal transduction in differentiating chondrocytes is also unknown. The increased trabecular bone formation in the epiphysis of the *Cldn12* KO mice is unlikely the result of altered intestinal absorption or renal reabsorption because lack of CLDN 12 alone did not affect urinary or fecal excretion of calcium (27). In other cell types, several TSPAN family members have been shown to interact with CLDN proteins to promote Notch signaling (33). Future studies are needed to determine if there is an interaction of CLDN12 with TSPAN proteins in bone and how this interaction would promote cellular signaling in differentiating articular chondrocytes.

## Data Availability Statement

The raw data supporting the conclusions of this article will be made available by the authors, without undue reservation.

## Ethics Statement

The animal study was reviewed and approved by the Institutional Animal Care and Use Committee of the Jerry L. Pettis Memorial Veterans Affairs Medical Center.

## Author Contributions

Conceptualization, SM. Methodology, YC. Data curation, SP, YC, and WX. Writing-original draft preparation, WX and SM. Writing, review and editing, WX and SM. Supervision, SM. Funding acquisition, SM. All authors have read and agreed to the published version of the manuscript.

## Funding

This work was supported by National Institutes of Health grant R01 AR070806 (to SM). The Department of Veterans Affairs in Loma Linda, California provided facilities to carry out this work. SM is a recipient of Senior Research Career Scientist Award from the Veterans Administration.

## Conflict of Interest

The authors declare that the research was conducted in the absence of any commercial or financial relationships that could be construed as a potential conflict of interest.

## Publisher’s Note

All claims expressed in this article are solely those of the authors and do not necessarily represent those of their affiliated organizations, or those of the publisher, the editors and the reviewers. Any product that may be evaluated in this article, or claim that may be made by its manufacturer, is not guaranteed or endorsed by the publisher.
